# A YOLO-Based Model for Detecting Stored-Grain Insects on Surface of Grain Bulks

**DOI:** 10.3390/insects16020210

**Published:** 2025-02-14

**Authors:** Xueyan Zhu, Dandan Li, Yancheng Zheng, Yiming Ma, Xiaoping Yan, Qing Zhou, Qin Wang, Yili Zheng

**Affiliations:** 1 School of Technology, Beijing Forestry University, Beijing 100083, China; xueyan0111@bjfu.edu.cn (X.Z.); dandanli2011@126.com (D.L.); 2 Sinograin Chengdu Storage Research Institute Co., Ltd., Chengdu 610091, China; 3 China Reserve Grain Management Group Co., Ltd., Beijing 100044, China; 4 Chengdu Sinograin Reserves Co., Ltd., Chengdu 610073, China; 5 National Key Laboratory-Forest Resource Efficient Production, Beijing 100083, China; 6 Key Laboratory of National Forestry and Grassland Administration on Forestry Equipment and Automation, Beijing 100083, China

**Keywords:** stored-grain insect, integrate pest management, tiny object detection, grain bulks surface, YOLO-SGInsects

## Abstract

Accurate, rapid, and intelligent stored-grain insect detection and counting are important for integrated pest management (IPM). Existing stored-grain insect pest detection models are often not suitable for detecting tiny insects on the surface of grain bulks and often require high computing resources and computational memory. Therefore, this study presents a YOLO-SGInsects model based on YOLOv8s for tiny stored-grain insect detection on the surface of grain bulk by adding a tiny object detection layer (TODL), adjusting the neck network with an asymptotic feature pyramid network (AFPN), and incorporating a hybrid attention transformer (HAT) module into the backbone network. The YOLO-SGInsects model was trained and tested using a GrainInsects dataset with images captured from granaries and laboratory. Experiments on the test set of the GrainInsects dataset showed that the YOLO-SGInsects achieved a stored-grain insect pest detection mean average precision (mAP) of 94.2%, with a counting root mean squared error (RMSE) of 0.7913, representing 2.0% and 0.3067 improvement over the YOLOv8s, respectively. Compared to other mainstream approaches, the YOLO-SGInsects model achieves better detection and counting performance and is capable of effectively handling tiny stored-grain insect pest detection in grain bulk surfaces. This study provides a technical basis for detecting and counting common stored-grain insect pests on the surface of grain bulk.

## Simple Summary

This study addresses the challenge of accurately and efficiently detect-ing tiny stored-grain insect pests on grain bulk surfaces, a critical task for integrated pest management (IPM). Existing detection models often struggle with small insects and require high computational resources. To overcome these limitations, the researchers developed YOLO-SGInsects, an enhanced YOLOv8s-based model incorporating a tiny-object detection layer, an asymptotic feature pyramid network, and a hybrid attention transformer module. Trained and tested on the GrainInsects dataset, which includes six insect species, the model achieved a mean average precision (mAP) of 94.2% and a counting root-mean-squared error (RMSE) of 0.7913, outperforming other mainstream detection models. The results demonstrate that YOLO-SGInsects can effectively detect and count tiny insects on grain surfaces, providing a valuable technical basis for improving IPM in granaries. This advancement has significant societal value as it enhances food security by enabling more effective pest control in grain storage facilities. Future research will focus on deploying the model on edge devices for mobile applications.

## 1. Introduction

Population growth has intensified the demand for food, making food conservation a central global concern. To address this increased food demand, it is necessary not only to enhance yields through improved seed and planting processes but also to reduce storage losses. Studies indicate that losses due to pest insects during grain storage account for 9 to 20 percent or more of total grain losses, particularly in developing countries [1,2]. These pests not only degrade both the quality and quantity of stored grain but also elevate the risk of fungal contamination [3]. Today, stored-grain pest control is a critical aspect of granaries management [4]. For stored-grain insect pests, early detection and prompt intervention are essential to reduce losses; therefore, continuous monitoring is critical to enable timely responses and minimize storage losses.

Visual inspection, probe sampling, and pest trap methods are the most popular conventional methods to detect stored-grain insects [5,6]. Among these methods, visual inspection is a consistent, qualitative, and subjective approach, commonly used as a standard for comparing quantitative methods. The probe sampling method involves probing grain from storage granaries, followed by sieving to separate pests [7]. By contrast, the trapping method involves placing traps at specific locations in the grain silo to detect stored-grain insects [8,9]. However, while relatively simple, the above conventional stored-grain insect detection methods are time-consuming and prone to subjectivity. Therefore, investigating fast and objective methods for automatically detecting stored-grain insects is essential.

To automatically detect stored-grain insect pests, researchers have explored the use of sensors [10,11], radiation sources [12,13], volatile compounds [14,15], and sound-based methods [16,17]. For example, Eliopoulos et al. [18] proposed a method based on bioacoustics to estimate the density of pests inside the stored-grain mass. Wu et al. [19] employed the potential of using AlphaMOS Fox3000 E-Nose to detect the stored-grain pests of red flour beetle (RFB) and rusty grain beetle (RGB) in stored grain. However, while the above methods are capable of detecting insects, they face challenges such as low accuracy and high costs, primarily due to their susceptibility to environmental noise.

Recently, with advancements in computer vision, numerous researchers have explored the use of object detection algorithms in computer vision to detect stored-grain insect pests [20,21]. For instance, Zhao et al. [22] proposed an enhanced YOLOv7 (You Only Look Once version 7) model to detect stored-grain insect pests. In their approach, Convolutional Block Attention Module (CBAM), Mixed Self-Attention and Convolution (ACmix), and Efficient Complete Intersection over Union (ECIoU) were combined with YOLOv7, resulting in an impressive mean average precision (mAP) and an F1 score of 91.9% and 89.6%, respectively. Similarly, Chen et al. [23] proposed a stored-grain insect pest detection method based on YOLOv4. Experimental results demonstrated that their method achieved a mAP of 97.55%. Shi et al. [24] presented a stored-grain insect pest detection and identification method based on R-FCN and achieved a mAP of 88.06% when evaluated on a test dataset. Yu et al. [25] developed a novel CACNet model for stored-grain insect pest detection and control in stored grain, which achieved a structure measure of 91.9% and a weighted F-measure of 76.4%. Shen et al. [26] collected stored-grain insect image data using an online insect trapping device and used an optimized Faster RCNN for detection and counting of six species of common stored-grain insect pests, achieving a mAP of 88%. However, most existing studies focus solely on detecting insects in specific types of grain, with limited research addressing the detection of multiple insects across multiple grain types. Furthermore, existing methods inadequately address the challenge of detecting small insects in stored grains.

In granaries, stored-grain insect pests in grain bulks are typically small, posing challenges for detection [27]. Additionally, stored-grain insect pests often hide in grain bulks or inside grain kernels, further complicating detection [28]. As a result, designing a specialized model to address the small-object issue in stored-grain insect pest detection within grain bulks is essential for improving detection accuracy. In this study, the YOLOv8s was chosen as the base model for insect detection due to its high inference speed and efficiency. Its anchor-free detection head and enhanced feature extraction capabilities make it well-suited for accurate and efficient detection in granary environments.

**Figure 1:**
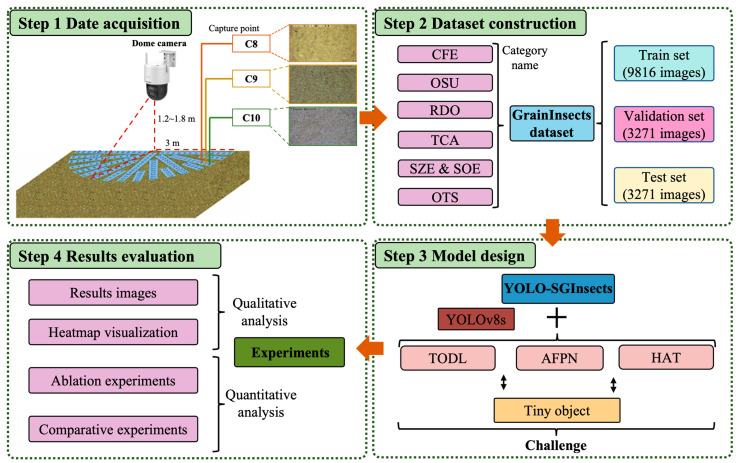
Overall workflow of proposed stored-grain insect pest detection methods. CFE: rusty grain beetle (*Cryptolestes ferrugineus* (Stephens)), OSU: sawtoothed grain beetle (*Oryzaephilus surinamensis* (Linnaeus)), RDO: lesser grain borer (*Rhyzopertha dominica* (Fabricius)), TCA: red flour beetle (*Tribolium castaneum* (Herbst)), SZE: maize weevil (*Sitophilus zeamais* (Linnaeus)), SOE: rice weevil (*Sitophilus oryzae* (*Motschulsky*)), OTS: insects with categories not identified by experts resulted by shooting defects, low resolution, or insufficient exposure. TODL: tiny object detection layer, AFPN: asymptotic feature pyramid network, HAT: hybrid attention transformer module.

In this study, a novel YOLOv8s-based model for detecting stored-grain insect pests on the surface of grain bulks is developed, which can be used to detect multiple insects across multiple grain types. This method improves the detection accuracy of stored-grain insect pests on the surface of grain bulks. Figure 1 presents the workflow of the stored-grain insect detection methods. The main contributions of this paper are as follows:

(1) A stored-grain insect pest detection dataset (GrainInsects) was constructed covering six stored-grain insect pest species.
(2) A novel YOLO-SGInsects model was developed for stored-grain insect pest detection and counting based on YOLOv8s, which improves recognition of tiny stored-grain insect pests in grain bulks.

## 2. Materials and Methods

### 2.1. Data Collection and Dataset Construction

**Figure 2:**
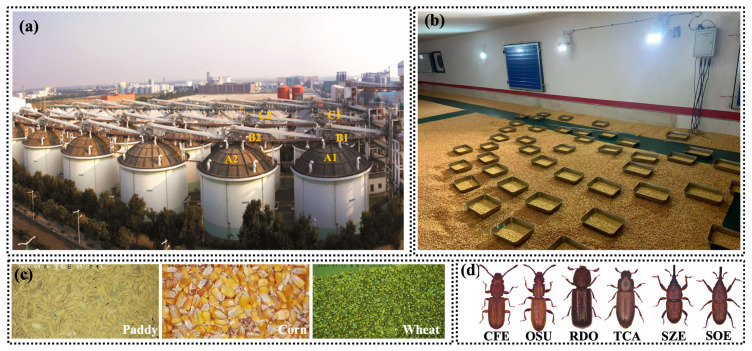
Experiment granary, interior view of the granary, images of typical cereals, and images of typical stored-grain insect pests. (**a**) Experiment granary for stored-grain insect pest image data collection (paddy: A1,A2; corn: B1,B2; wheat: C1,C2); (**b**) interior view of granary; (**c**) images of three typical cereals; and (**d**) images of typical stored-grain insects. CFE: rusty grain beetle (*Cryptolestes ferrugineus* (Stephens)), OSU: sawtoothed grain beetle (*Oryzaephilus surinamensis* (Linnaeus)), RDO: lesser grain borer (*Rhyzopertha dominica* (Fabricius)), TCA: red flour beetle (*Tribolium castaneum* (Herbst)), SZE: maize weevil (*Sitophilus zeamais* (Linnaeus)), and SOE: rice weevil (*Sitophilus oryzae* (*Motschulsky*)).

In this study, typical stored-grain insect pests commonly found in paddy, corn, and wheat were selected as research subjects. Image data were collected using dome cameras in the grain bins and laboratories of the China Grain Reserve Management Group Corporation (CGRC) directly affiliated grain depots. Six grain depots (paddy: A1,A2; corn: B1,B2; wheat: C1,C2)were selected for data collection, with two bins allocated to each type of grain (e.g., Figure 2a). To address the limited number of insects naturally present in the grain bins and enrich the data, trays containing grain were placed under the dome camera, and insects were introduced in appropriate numbers for image acquisition (e.g., Figure 2b). The data acquisition method in the laboratory environment was similar to that used in the grain bins and involved photographing grain trays containing introduced insects. Typical images of grain surfaces captured by the dome camera are shown in Figure 2c. Figure 2d depicts several common stored grain insects found in paddy, corn, and wheat.

A total of 16,358 insect images were ultimately collected including the six typical stored-grain insects indicated above. Among them, 5123 images used naturally grown insects in granaries, 2887 images used the introduced insects in trays in granaries, and 8348 images used introduced insects in trays in the laboratory. Due to variations in the occurrence frequency and activity levels of different insect species, the collected quantities of insects vary significantly among species. Additionally, the number of insects within a single image also differs. Therefore, this study does not distinguish between grain types or insect species but instead treats all insects as a single category for analysis.

Five experienced operators conducted data labeling using LabelImg software (version 1.8.3, available at https://github.com/tzutalin/labelImg, accessed on 5 October 2015). LabelImg is an open-source tool designed for annotating objects of interest, with support for exporting data in multiple formats suitable for training diverse object detection models. During stored-grain insect pest image labeling, based on expert experience and practical needs, only insects with the whole body fully exposed, with half the body and antennae exposed, and with half the body and feet exposed were labeled. To ensure accuracy, stored-grain insect pest images labeled by the five grain managers underwent cross-checking, with final labeling decisions made collaboratively to resolve any uncertainties. Ultimately, a total of 66,372 stored-grain insect pests were labeled. It is worth noting that the manually annotated insects include not only the six species mentioned above but also some insects whose characteristics are insufficiently discernible due to issues such as imaging defects, resolution limitations, or occlusion, making accurate identification of the species difficult. The labeled stored-grain insect pest images served as the foundation for constructing the grain bulk surface insect pest detection dataset (GrainInsects) [29]. Following a 6:2:2 ratio, 16,358 stored-grain insect pest images, containing 66,372 labeled insect pests, were divided into training, validation, and test datasets. Table 1 provides detailed information on the stored-grain insect pests detection dataset.

### 2.2. Origional YOLOv8s Model

**Figure 3:**
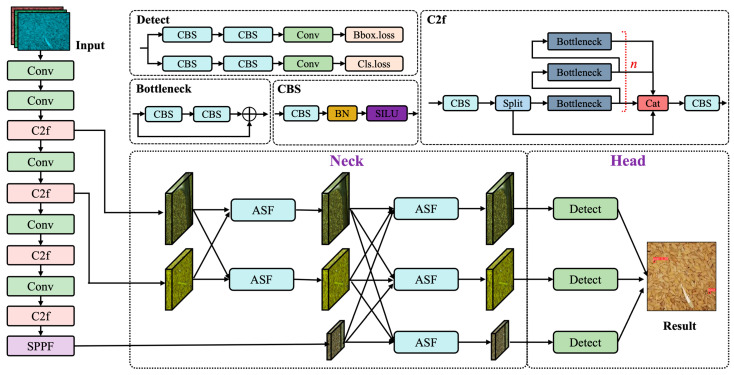
Network structure of YOLOv8s. Conv: convolutional layer, C2f: CSP Bottleneck with two convolutions, SPPF: Spatial Pyramid Pooling-Fast, CBS: combination of Convolution, Batch Normalization, and SiLU activate function, BN: Batch Normalization, Cat: Concatenate, SILU: SiLU activate function, ASF: adaptive spatial fusion.

The development of the stored-grain insect pest detection model involved selecting a suitable baseline model through a comparative analysis of widely used object detection frameworks. The models analyzed included one-stage detectors such as YOLOv5s, YOLOv6s [30], YOLOv7-tiny [31], YOLOv8s [32], and RT-DETR [33], along with the two-stage detector Faster RCNN [34]. The evaluation focused on models that achieved an optimal balance between accuracy and computational efficiency. Within the YOLO series, lightweight versions with reduced parameters, model size, and FLOPs were specifically selected to ensure applicability in resource-limited environments. Based on this analysis, YOLOv8s emerged as the most suitable base model for further development, offering an excellent trade-off among precision, speed, and adaptability. Figure 3 illustrates the network structure of YOLOv8s, which consists primarily of three components: the backbone network, the neck network, and the head network. The backbone network performs multi-scale feature extraction of the input image, while the neck network uses a feature pyramid structure to integrate feature maps from different scales and levels, thereby improving detection accuracy. The head network generates the final detection results by processing features extracted at various scales and levels.

### 2.3. YOLO-SGInsects Model

**Figure 4:**
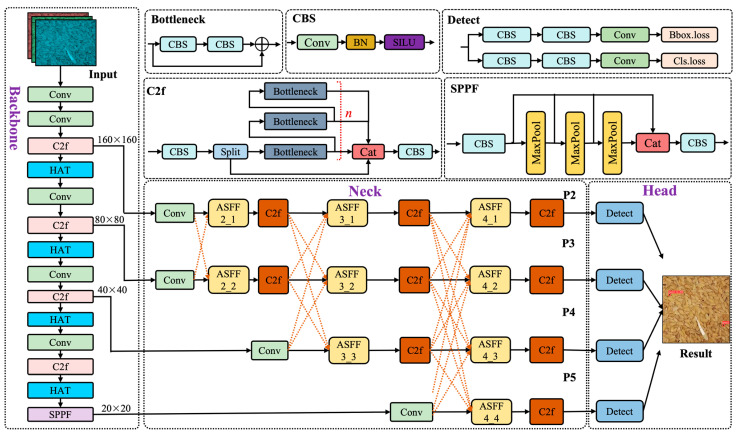
Network structure of YOLO-SGInsects model based on modified YOLOv8s. HAT: hybrid attention transformer module, ASFF: Adaptively Spatial Feature Fusion, Conv: convolutional layer, C2f: CSP Bottleneck with two convolutions, SPPF: Spatial Pyramid Pooling-Fast, CBS: combination of Convolution, Batch Normalization, and SiLU activate function, BN: Batch Normalization, Cat: Concatenate, SILU: SiLU activate function.

To improve the detection accuracy of stored-grain insect pests, the YOLOv8s-based stored-grain insect pest detection model (YOLO-SGInsects) was developed with three key enhancements. Specifically, the backbone network, neck network, and head network of YOLOv8s were modified. First, a dedicated tiny object detection layer (TODL) was designed to improve the model’s ability to detect small stored-grain insects. Next, the asymptotic feature pyramid network (AFPN) was adopted to construct the neck network, enabling the adaptive fusion of multi-level feature maps to effectively capture contextual information at different scales and address the challenge of detecting small stored-grain insect pests. Third, the hybrid attention transformer (HAT) was incorporated into the backbone network to enhance the visibility of small objects by increasing the resolution of the feature map, thereby improving the stored-grain insect pest detection performance. Figure 4 illustrates the network structure of the YOLO-SGInsects model.

#### 2.3.1. Tiny Object Detection Layer

The original YOLOv8s model consists of three detection heads, P3, P4, and P5, which use feature maps of 80 × 80, 40 × 40, and 20 × 20 to detect large (32 × 32), medium (16 × 16), and small (8 × 8) objects, respectively. However, for detecting stored-grain insect pests on grain bulk surfaces, satisfactory results are difficult to achieve with the above three detection heads in YOLOv8s. This difficulty arises mainly due to the relatively large down-sampling multiplier in YOLOv8s, which limits the learning of tiny object features from deeper feature maps. Previous studies indicate that specialized tiny-object detection heads effectively enhance the detection capability for tiny objects [35,36]. To address this challenge, a specialized tiny object detection head was incorporated into YOLOv8s. Figure 4 illustrates the YOLO-SGInsects model with an additional tiny object detection layer (P2).

As shown in Figure 4, the YOLO-SGInsects model includes an additional output layer, P2, alongside the existing output layers P3, P4, and P5, enhancing the detection ability of stored-grain insect pests on grain bulk surfaces. In addition, the feature map of output layer P2 is 160 × 160, allowing the detection of stored-grain insect pests as small as 4 × 4 pixels.

#### 2.3.2. Asymptotic Feature Pyramid Network

**Figure 5:**
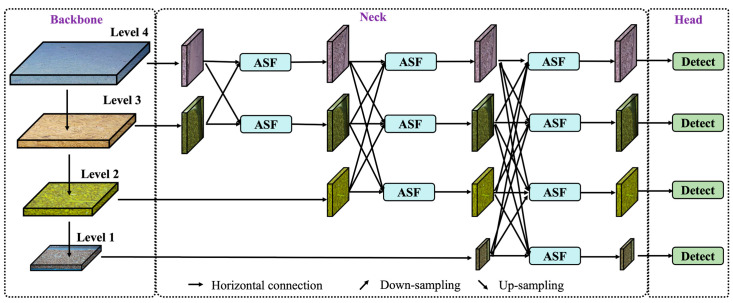
Network structure of asymptotic feature pyramid module. ASF: adaptive spatial fusion.

The YOLOv8s model combines feature maps of different levels through a bottom-up feature pyramid network, enabling the detection of objects of varying sizes within an image. However, previous studies indicate that this traditional feature pyramid network can suffer from feature information loss or degradation and conflicts, particularly in the fusion of non-adjacent layers [37]. These issues impact detection accuracy, especially for tiny objects like stored-grain insect pests on grain bulk surfaces, which are significantly difficult to detect. To address this challenge, the AFPN module was introduced into the YOLOv8s model, progressively fusing features from different layers to enhance the detection of tiny objects like stored-grain insect pests on grain bulk surfaces. The network structure of the AFPN module is shown in Figure 5.

As shown in Figure 5, AFPN incrementally integrates low-level, high-level, and top-level features into the stored-grain insect pests detection process using a gradual feature fusion strategy. This hierarchical fusion helps to effectively utilize semantic information across levels and reduces the semantic gap between feature levels during fusion, enabling the model to fully understand and leverage multi-level features and improving feature fusion quality. Additionally, AFPN incorporates an adaptive spatial fusion mechanism to emphasize key information during feature fusion while mitigating the influence of conflicting information. This progressive fusion effectively minimizes the semantic gap across feature levels and enhances fusion quality, enabling the YOLO-SGInsects model to adapt more effectively to multi-level semantic information in the stored-grain insect pest detection on grain bulk surface.

#### 2.3.3. Hybrid Attention Transformer Module

**Figure 6:**
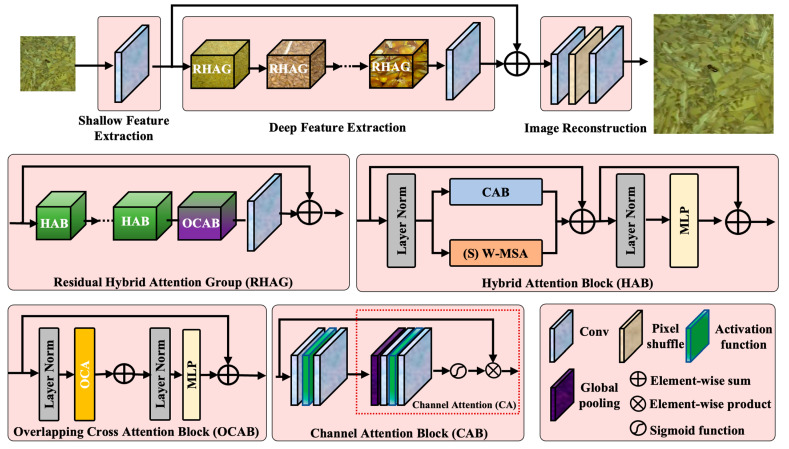
Structure of hybrid attention transformer module. RHAG: residual hybrid attention group, HAB: hybrid attention block, OCAB: overlapping cross attention block, CAB: channel attention block, W-MSA: window-based multi-head self-attention, OCA: overlapping cross attention, MLP: Multi-Layer Perceptron, Conv: convolutional layer.

Strengthening the feature fusion ability of the feature pyramid network (FPN) of YOLOv8s can help improve the detection performance of small stored-grain insect pests. The HAT module proposed by Chen et al. [38] is an attention mechanism based on super-resolution reconstruction that aims to enhance the visibility of small objects by increasing the resolution of the feature map. It achieves higher feature map resolution while maintaining a lightweight model, thereby improving small-object detection accuracy through the integration of a super-resolution reconstruction module [39,40]. Moreover, the HAT module enhances the model’s sensitivity to small objects while minimizing the additional computational cost compared to traditional attention mechanisms. The HAT module refines the feature representation of stored-grain insect pest regions through a super-resolution reconstruction process, thereby enhancing the model’s ability to detect stored-grain insects. Specifically, this study integrates HAT modules at each level of the FPN to strengthen the representation of multi-scale features. Figure 6 presents the detailed structure of the HAT module.

As shown in Figure 6, the HAT module comprises three components: shallow feature extraction, deep feature extraction, and image reconstruction. The shallow feature extraction component primarily consists of convolutional layers and is responsible for extracting the shallow features of the input image. The deep feature extraction component comprises multiple residual hybrid attention groups (RHAGs) and 3 × 3 convolutional layers, designed to extract the deep features of the image. The image reconstruction component consists of convolutional blocks and a pixel shuffle module, tasked with improving resolution and generating the output image.

The RHAG consists of multiple hybrid attention block (HAB) layers, an overlapping cross attention block (OCAB), and a 3 × 3 convolutional layer with residual concatenation, specifically designed to enhance feature extraction and image reconstruction. The HAB comprises two components: a window-based self-attention mechanism and a channel attention mechanism. When the input feature map enters the HAB, it undergoes normalization before being processed by the window-based self-attention mechanism. Specifically, the window-based self-attention mechanism partitions the feature map into multiple localized windows and computes self-attention within each window to capture correlation information within localized regions. Subsequently, global features are introduced through the channel attention mechanism, which computes attention weights across channels. This mechanism applies attention weights to features by leveraging global insights, effectively activating pixels relevant to the image.

In addition, the OCAB enhances the network’s feature representation capability by incorporating a cross attention layer that establishes connections across windows based on window self-attention. The overall architecture combines the strengths of local and global feature representations, resulting in substantial improvements in image reconstruction performance.

### 2.4. Experimental Platform and Configuration

All models (including: YOLOv5s, YOLOv6s, YOLOv7-tiny, YOLOv8s, RT-DETR, Faster RCNN, YOLOv8s, and YOLO-SGInsects) used in this study were trained and tested on the same server. Table 2 outlines the hardware and software configurations of the server. For training the YOLO-SGInsects model, the input image size was set to 640 × 640, with a batch size of 32, an epoch of 500, a learning rate of 0.01, and a weight decay coefficient of 0.0005. Furthermore, the hyperparameter settings for the other models in the experiment were aligned with those of YOLO-SGInsects. To accelerate model training, transfer learning was employed, initializing the parameters with weights pre-trained on the ImageNet dataset.

### 2.5. Model Evaluation

To comprehensively evaluate the effectiveness of the model in stored-grain insect pest detection, the precision (*P*), recall (*R*), and mean average precision (mAP) were selected as key evaluation metrics [25,41]. The precision represents the proportion of positive samples predicted by the model that are truly positive, while the recall measures the proportion of actual positive samples correctly identified by the model. The mAP provides a comprehensive assessment of the model’s effectiveness across various stored-grain insect species. All three metrics—precision, recall, and mAP—range between 0 and 1, with higher values indicating better detection performance and lower values suggesting poorer performance. In addition to these accuracy measures, the number of parameters, number of floating-point operations, and layer number were also recorded to provide a more comprehensive evaluation of the different stored-grain insect pest detection models. Equations (1)–(3) present the calculation formula of *P*, *R*, and mAP.

(1) $$P=\frac{T_{P}}{T_{P}+F_{P}}$$

(2) $$R=\frac{T_{P}}{T_{P}+F_{N}}$$

(3) $$mAP=\frac{\int_{0}^{1}P\cdot RdR}{M}$$

where *T_P_* denotes the number of stored-grain insects that were correctly detected, *F_P_* denotes the number of objects incorrectly detected, and *F_N_* denotes the number of stored-grain insects not detected. *M* denotes the number of categories for detection. In this study, all stored-grain insect pest categories were grouped as a single category, setting *M* to 1.

To evaluate the performance of different models in counting stored-grain insects on the surface of grain bins, this study selected three quantitative indicators: mean absolute error (MAE), root mean squared error (RMSE), and coefficient of determination (*R*^2^). The formulas for MAE, RMSE, and *R*^2^ are as follows:

(4) $$MAE=\frac{1}{n}\sum_{i=1}^{n}\left|y_{i}-{\hat{y}}_{i}\right|$$

(5) $$RMSE=\sqrt{\frac{1}{n}\sum_{i=1}^{n}{\left(y_{i}-{\hat{y}}_{i}\right)}^{2}}$$

(6) $$R^{2}=1-\frac{\sum_{i=1}^{n}{\left(y_{i}-{\hat{y}}_{i}\right)}^{2}}{\sum_{i=1}^{n}{\left(y_{i}-{\bar{y}}_{i}\right)}^{2}}$$

where *n* represents the total number of test images and *i* represents the *i*th test image. *y_i_* represents the actual number of stored-grain insects in the *i*th image, ${\hat{y}}_{i}$ represents the number of stored-grain insects in the *i*th image as predicted by the model, and $\bar{y}$ represents the mean of the actual number of stored-grain insects across all *n* images.

In addition to the quantitative evaluation presented above, this study employed Grad-CAM to qualitatively assess the detection outcomes of stored-grain insects. Grad-CAM is a visualization technique that calculates the significance of spatial locations in the convolutional layers using gradients, providing valuable insights into the regions the model focuses on during decision making [42]. Visualizing these regions allowed us to identify the areas of the input images that contributed most to the model’s predictions, offering a clearer understanding of its behavior and enabling further analysis of model performance.

## 3. Results and Discussion

### 3.1. Stored-Grain Insect Pest Detection and Counting Results

**Figure 7:**
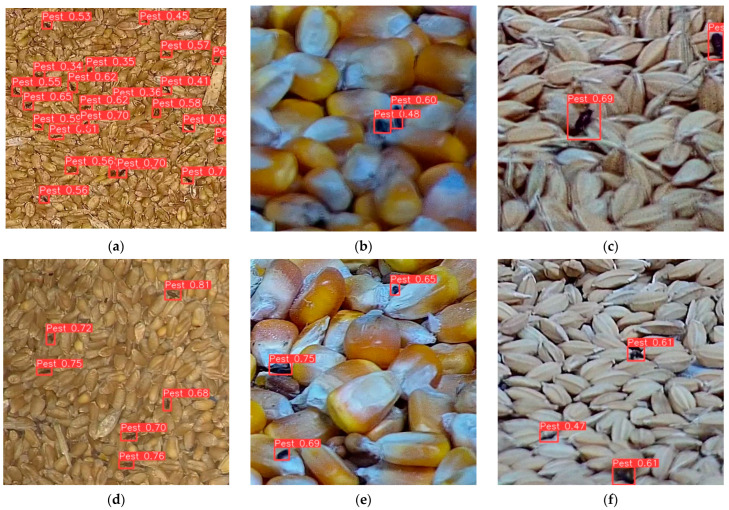
Example of typical stored-grain insect pest detection results using YOLO-SGInsects in (**a**,**d**) wheat, (**b**,**e**) corn, and (**c**,**f**) paddy. The number in red color bar on top of each insect is the confidence of each insect detected by YOLO-SGInsects.

Experiment results indicated that the YOLO-SGInsects model successfully detected stored-grain insect pests on the surface of the grain bulk, achieving a precision of 89.7%, a recall of 90.1%, and a mAP@0.5 of 94.2%. Additionally, YOLO-SGInsects accurately counted the number of stored-grain insect pests in the grain bulk surfaces, with MAE, RMSE, and *R*^2^ values of 0.2582, 0.7913, and 0.9546, respectively. Furthermore, the YOLO-SGInsects model maintained relatively high efficiency while keeping a manageable model parameter (31.37 M) and FLOPs (42.5 G). As illustrated in Figure 7, the YOLO-SGInsects model effectively achieved accurate detection of stored-grain insect pests on the surface of grain bulks, performing well across all three crops: wheat, corn, and paddy.

### 3.2. Ablation Experiments and Analysis of Each Function Module Within YOLO-SGInsects

In YOLO-SGInsects, three components were designed: the tiny object detection layer, the asymptotic feature pyramid module, and the contextual transformer module. To evaluate the contribution of each functional module to the YOLO-SGInsects model, an ablation experiment was conducted to focus on the TODL, AFPN, and HAT modules. Table 3 presents the results of the ablation experiments. In Table 3, ✗ indicates that the improvement strategy was not utilized, while ✓ indicates that the improvement strategy was employed.

Ablation experiments in Table 3 provided insights into the importance of TODL, AFPN, and HAT modules in the YOLO-SGInsects model. By comparing the evaluation metrics in Table 3, the variation of precision, recall, and mAP@0.5 could be observed when removing the TODL, AFPN, and HAT modules. The ablation experiment and analysis were helpful in fully understanding the contribution and effectiveness of TODL, AFPN, and HAT modules in YOLO-SGInsects.

Table 3 demonstrated that the three improvement strategies effectively enhanced the accuracy of stored-grain pest detection on the surface of grain bulk. Specifically, the precision, recall, and mAP@0.5 values of YOLOv8s for stored-grain insect pest detection on grain bulk surface were 84.8%, 88.5%, and 92.2%, showed a higher rate of false detections. After adding TODL into YOLOv8s, the precision, recall, and mAP@0.5 improved to 87.3%, 89.9%, and 93.9%, respectively, representing enhancements of 2.5%, 1.4%, and 1.7% compared to YOLOv8s. Additionally, when combining YOLOv8s with the AFPN module, the precision, recall, and mAP@0.5 for stored-grain insect pest detection on the surface of store-grain bulks improved to 86.9%, 89.7%, and 93.6%, respectively. Furthermore, the combination of YOLOv8s with the HAT attention module also enhanced the detection of stored-grain insect pests on the surface of grain bulks.

When the TODL, AFPN, and HAT attention modules were simultaneously integrated into YOLOv8s to create the YOLO-SGInsects model, its precision, recall, and mAP@0.5 reached 89.7%, 90.1%, and 94.2%, significantly outperforming other methods in the ablation experiment. Furthermore, the model parameters and FLOPs of YOLO-SGInsects remained comparable to those of the YOLOv8s. The results of the ablation experiments demonstrated that the YOLO-SGInsects model achieved improved stored-grain insect pest detection accuracy while maintaining a similar number of parameters.

### 3.3. Visualization of Gradient Heatmaps

**Figure 8:**
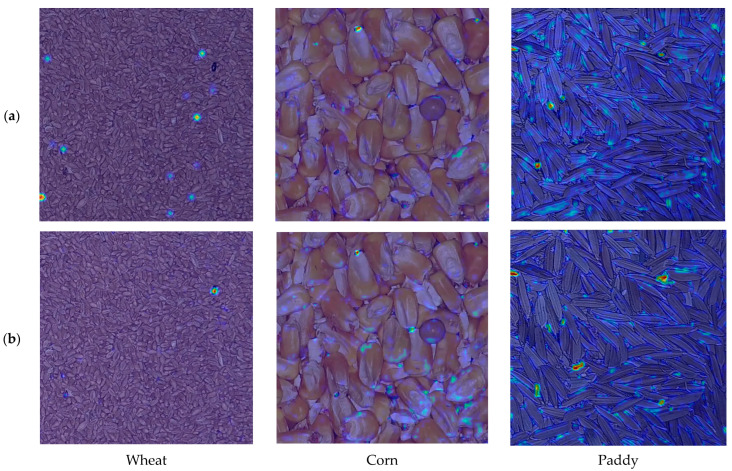
Comparison of gradient heatmaps between YOLOv8s and YOLO-SGInsects. (**a**) YOLOv8s; (**b**) YOLO-SGInsects. Darker reddish areas indicate a greater contribution of those regions to stored-grain insect pest detection decision.

Figure 8 presented the heat map visualization results of YOLOv8s and YOLO-SGInsects using Grad-CAM. As illustrated in Figure 8, the YOLO-SGInsects model outperformed YOLOv8s in detecting stored-grain insect pests on the surface of grain bulks. Specifically, YOLO-SGInsects concentrated more attention on stored-grain insect pests during detection, with minimal attention on backgrounds. While YOLOv8s could localize stored-grain insect pest areas, its attention is not as concentrated as that of YOLO-SGInsects. In contrast, YOLOv8s was easily affected by complex backgrounds. The gradient heatmap comparison revealed that the YOLO-SGInsects model more accurately located the stored-grain insect pests on the surface of the grain bulk image while effectively resisting interference from complex backgrounds, thus confirming the effectiveness of the improvements.

### 3.4. Comparison and Analysis with Mainstream Object Detection Models

To validated the performance of YOLO-SGInsects in detecting and counting stored-grain insect pests, we compared it with other commonly used mainstream object detection models. The same test datasets captured from the actual environments were input into the above models, and the evaluation results are presented in Table 4.

Table 4 illustrated the quantitates results of various models for detecting stored-grain insect pests on the surface of grain bulks in the comparative experiments. By thoroughly analyzing the outputs of the comparison experiment, we concluded that the YOLO-SGInsects model surpassed the mainstream object detection models in terms of precision, recall, and mAP, particularly for the detection of stored-grain insect pests on the surface of grain bulks. Specifically, the detection mAP of YOLO-SGInsects reached 94.2%, represented an improvement of 3.7%, 2.6%, 2.8%, 2.3%, 2.1%, and 2.0% over Faster RCNN, RT-DETR, YOLOv5s, YOLOv6s, YOLOv7-tiny, and YOLOv8s, respectively. However, it is worth noting that the YOLO-SGInsects model has larger parameters and FLOPs than YOLOv5s, YOLOv6s, YOLOv7-tiny, and YOLOv8s, which represent areas for future improvements and optimization.

**Figure 9:**
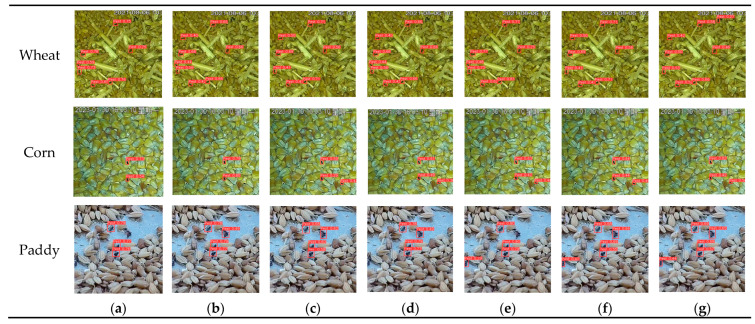
Example images of insect detection results using different models. (**a**) Faster RCNN; (**b**) RT-DETR; (**c**) YOLOv5s; (**d**) YOLOv6s; (**e**) YOLOv7-tiny; (**f**) YOLOv8s; and (**g**) YOLO-SGInsects.

Figure 9 presented examples of detection and counting results for various models based on test dataset images in the comparative experiment. The effectiveness of YOLO-SGInsects for detecting stored-grain insect pests on the surface of grain bulks was superior to that of the other methods. Specifically, the Faster RCNN, RT-DETR, YOLOv5s, YOLOv6s, YOLOv7-tiny, and YOLOv8s models exhibited varying degrees of false and missed detections, while YOLO-SGInsects achieved accurate stored-grain insect pest detection on the surface of grain bulks, with minimal false negatives or positives. Comparative experiments result demonstrated that the YOLO-SGInsects model is effective in detecting stored-grain insect pests on the surface of grain bulks.

## 4. Conclusions

For the automatic and accurate detection of stored-grain insect pests on the surface of grain bulk, a novel YOLOv8s-based stored-grain insect pests detection model (YOLO-SGInsects) based on YOLOv8s has been developed in the study. The proposed method significantly enhanced the stored-grain insect pest detection performance on grain bulk surfaces by introducing a tiny object detection layer, AFPN, and HAT attention module to YOLOv8s. Experimental results demonstrated that the YOLO-SGInsects model outperformed mainstream object detection models (Faster RCNN, RT-DETR, YOLOv5s, YOLOv6s, YOLOv7-tiny, and YOLOv8s), achieving the highest accuracy (mAP = 94.2%). Moreover, the gradient heatmaps comparison also demonstrated that the YOLO-SGInsects model focused more on stored-grain insect pest regions, which is essential for stored-grain insect pest detection on the surfaces of grain bulks. These results demonstrate that the YOLO-SGInsects model for automatic stored-grain insect pest detection on the surfaces of grain bulk potentially provide a reference method for integrated pest management in granaries. Further research is required to deploy the model to edge devices that match the requirements for onsite mobile platform applications.

## Figures and Tables

**Table 1:** Composition details of stored-grain insect pest detection dataset (GrainInsects).

Dataset	Image Number	Stored-Grain Insect Pest Number					
		CFE	OSU	RDO	TCA	SZE and SOE	OTS
Training set	9816	11,140	1596	7709	5054	11,833	1854
Validation set	3271	4164	409	2763	1600	3464	578
Test set	3271	4409	439	3160	1568	4027	605
Total	16,358	19,713	2444	13,632	8222	19,324	3037

Note: CFE: rusty grain beetle (*Cryptolestes ferrugineus* (Stephens)), OSU: sawtoothed grain beetle (*Oryzaephilus surinamensis* (Linnaeus)), RDO: lesser grain borer (*Rhyzopertha dominica* (Fabricius)), TCA: red flour beetle (*Tribolium castaneum* (Herbst)), SZE: maize weevil (*Sitophilus zeamais* (Linnaeus)), SOE: rice weevil (*Sitophilus oryzae* (*Motschulsky*)), OTS: insects with categories not identified by experts resulted by shooting defects, low resolution, or insufficient exposure.

**Table 2:** Detail of hardware and environment resource configuration.

Hardware	Configure	Hardware	Configure
CPU	Intel (R) Xeon (R) Gold 6240C	Operating system	Ubuntu 20.04
GPU	NVIDIA GeForce RTX 3090	Accelerated environment	CUDA 11.3 CUDNN 8.2.1
Graphic memory	24 G	Development environment	Visual studio code
RAM	96 G	Framework	PyTorch 2.0.1 Torchvision 0.13.1
Hard disk	31 T	Code language	Python 3.8.13

**Table 3:** Ablation experiment on major components in YOLO-SGInsects model on GrainInsects dataset.

YOLOv8s	TODL	AFPN	HAT	*P* (%)	*R* (%)	mAP (%)	MAE	RMSE	*R* ^2^	Layer	Para (M)	FLOPs (G)
✓	✗	✗	✗	84.8	88.5	92.2	0.3654	1.0980	0.9157	168	11.13	28.4
✓	✓	✗	✗	87.3	89.9	93.9	0.3116	1.0413	0.9224	207	10.63	36.6
✓	✗	✓	✗	86.9	89.7	93.6	0.3192	1.0579	0.9204	637	12.04	33.4
✓	✗	✗	✓	86.3	89.5	93.3	0.3370	1.0789	0.9175	2355	26.74	28.6
✓	✓	✓	✓	89.7	90.1	94.2	0.2582	0.7913	0.9546	3704	31.37	42.5

Note: TODL: tiny object detection layer, AFPN: asymptotic feature pyramid network, HAT: hybrid attention transformer, *P*: precision, *R*: recall, mAP: mean average precision, MAE: mean absolute error, RMSE: root mean squared error, *R*^2^: coefficient of determination, Layer: model layer, Para: model parameters, FLOPs: Floating Point Operations.

**Table 4:** Comparison experiment results of YOLO-SGInsects and mainstream stored-grain insect pest detection models.

Models	*P* (%)	*R* (%)	mAP (%)	MAE	RMSE	*R* ^2^	Layer	Para (M)	FLOPs (G)
Faster RCNN	83.4	86.0	90.5	0.4207	1.3127	0.8829	160	28.30	940.95
RT-DETR	84.1	87.3	91.6	0.4228	1.2774	0.8901	584	42.76	130.5
YOLOv5s	84.2	87.8	91.4	0.3980	1.2043	0.8989	157	7.02	15.8
YOLOv6s	84.5	88.5	91.9	0.3905	1.1931	0.9005	142	16.30	44.0
YOLOv7-tiny	85.2	87.3	92.1	0.3840	1.1478	0.9085	208	6.01	13.04
YOLOv8s	84.8	88.5	92.2	0.3654	1.0980	0.9157	168	11.13	28.4
YOLO-SGInsects	89.7	90.1	94.2	0.2582	0.7913	0.9546	3704	31.37	42.5

Note: *P*: precision, *R*: recall, mAP: mean average precision, MAE: mean absolute error, RMSE: root mean squared error, *R*^2^: coefficient of determination, Layer: model layer, Para: model parameters, FLOPs: Floating Point Operations.

## Data Availability

Data will be made available on request.
